# Impact of maternal nutritional literacy and feeding practices on the growth outcomes of children (6–23 months) in Gujranwala: a cross-sectional study

**DOI:** 10.3389/fnut.2024.1460200

**Published:** 2025-01-07

**Authors:** Aaiza Tahreem, Allah Rakha, Rimsha Anwar, Roshina Rabail, Cristina Maria Maerescu, Claudia Terezia Socol, Florin Leontin Criste, Gholamreza Abdi, Rana Muhammad Aadil

**Affiliations:** ^1^National Institute of Food Science and Technology, University of Agriculture, Faisalabad, Pakistan; ^2^Department of Genetics, Department of Animal Science and Technology, University of Oradea, Oradea, Romania; ^3^Department of Biotechnology, Persian Gulf Research Institute, Persian Gulf University, Bushehr, Iran

**Keywords:** malnutrition, IYCF, knowledge, practices, growth

## Abstract

**Introduction:**

Malnutrition contributes to approximately 45% of deaths among under 5 years children in low and middle-income countries. Poor maternal knowledge and failure to comply with recommended Infant and Young Child Feeding (IYCF) practices are known risk factors for malnutrition but there are inconsistencies in the literature. Therefore, this cross-sectional study of 100 mother–child pairs in district Gujranwala aimed to assess maternal nutritional literacy (MNL) and maternal feeding practices (MFP) and their ultimate impacts on child growth.

**Methods:**

A researcher administered questionnaire that was the combination of WHO model questionnaires and FAO Nutrition-related KAP model questionnaires was used to collect the data from mothers while and anthropometric measurements of children were taken by following the standard methods.

**Results:**

Results showed that 57% of mothers had average nutritional knowledge and feeding practices were not satisfactory. Only 12% of mothers-initiated breastfeeding within the first hour of birth, 7% of infants were exclusively breastfed, while 27.27% continued to be breastfed beyond 1 year. Complementary foods were timely introduced to 53% of infants, 47% received minimum meal frequency and 34% met the minimum dietary diversity criteria. Additionally, the consumption of unhealthy foods, sugar-sweetened beverages, and zero consumption of fruits and vegetables was practiced by 71, 23, and 27% of infants and young children, respectively. Prevalence of stunting, wasting, underweight and overweight was 19, 9, 12, and 12%, respectively. A non-significant association was observed between MNL and MFP with growth outcomes with a few exceptions. The odds of being wasted (*β* = 1.903, OR = 6.706, *p* < 0.05) and underweight (*β* = 1.732, OR = 5.654, *p* < 0.05) are higher among children who had vaginal birth. Higher odds of being stunted (*β* = 2.173, OR = 8.788, *p* < 0.05) were observed among those whose mothers had middle school education compared to those having higher education.

**Conclusion:**

Overall results indicated a dire need not only to create nutritional awareness among mothers in Gujranwala but also to provide a support system for mothers to help them implement standard feeding practices.

## 1 Introduction

The global burden of malnutrition is unacceptably high, which is impacting the social and economic development of countries. Its incidence differs usually according to ethnic, cultural, and socioeconomic characteristics. Low and middle-income countries (LMICs) are facing the double burden of malnutrition (DBM) i.e., children are at risk of either overnutrition or undernutrition. Worldwide, approximately 45% of deaths among children under 5 years are attributed to undernutrition. UNICEF reported that in the year 2020, worldwide one in every five children had stunted growth accounting for 22% of children under 5 years. Moreover, 6.7% of children were wasted, 2% were severely wasted and 5.7% were overweight. In South Asia, the proportion of stunted, wasted, and overweight children is 31.8, 14.7, and 2.2%, respectively (1). DBM is becoming increasingly apparent in Pakistan; as per the National Nutritional Survey-2018 (NNS), the proportion of stunted, wasted, underweight, and overweight under 5 years children are 40.2, 17.7, 28.9, and 9.5%, respectively (2). Infant malnutrition has been linked to numerous consequences like increased frequency and severity of infections, delayed recoveries, increased energy requirements, and suppressed appetite and malabsorption (3). Malnutrition also results in delayed cognitive development affecting academic achievements, reduction in productivity and loss of country’s gross domestic product each year (1, 3, 4).

Progress has been made but still, it’s a long road to meet the global nutrition targets in 2025 for improving the health of mothers, infants, and young children. Many LMICs seem to miss the targets of reducing under 5-year stunting by 40%, maintaining and reducing childhood waste to not more than 5%, and no more increase in overweight children (5). For achieving the global targets, adequate Infant and Young Child Feeding (IYCF) practices are a must as the first 1,000 days of life are paramount. The first 1,000 days including pregnancy and the first 2 years of life offer a unique window of opportunity as 1,000 days include three crucial life stages: pregnancy, infancy, and toddlerhood. Nutrition is an indispensable fuel as it’s a period of critical brain development and a foundation for lifelong health. The provision of key nutrients during this period helps in sustaining and enhancing the nutritional status of children during their early stages of life. In addition, IYCF is essential for improving child survival, promoting healthy growth and development, and reducing the risk of chronic diseases. Thus, it plays a crucial role in building better and more productive communities in all walks of life (6). Globally, three out of every five newborns are not breastfed within the first hour of life with only about 44% of exclusively breastfeeding from 2014 through 2019. The proportion achieving MMF, MDD and MAD was 55, 28, and 18%, respectively (7, 8). According to NNS, nearly 45.8% of Pakistani newborns fulfill the criteria for early initiation of breastfeeding accounting for 37.7% exclusively breastfed children in 2011, which fortunately increased to 48% in 2018. The percentage of children receiving MMF, MDD, and MAD was 18.2, 14.2, and 3.6%, respectively. The situation in rural areas was even worse (2).

The IYCF goals set by WHO are adversely affected by insufficient nutrition-related knowledge, maternal health issues, breast milk insufficiency, work-related issues, lack of social support, and low socio-economic status. MNL has long been linked to child’s nutritional outcomes (9). Many studies have highlighted mothers’ suboptimal knowledge and practices in terms of breastfeeding and complementary feeding (10–13). The WHO and UNICEF have recommended the IYCF indicators as nutrition-specific parameters for monitoring dietary intake and guiding the development of appropriate interventions against child malnutrition in developing countries (14). Some additional indicators were introduced in 2021 but there are limited studies available involving all those indicators in terms of assessing their impact on malnutrition. Therefore, a cross-sectional survey-based study including 16 out of 17 indicators was conducted to investigate the impact of maternal nutritional literacy and feeding practices on the growth outcomes of infants and young children (6–23 months) in Gujranwala. The findings of this research will be helpful in better understanding the effective way of designing nutrition education programs to make the most out of these interventions.

## 2 Materials and methods

### 2.1 Study setting and population

**Figure 1:**
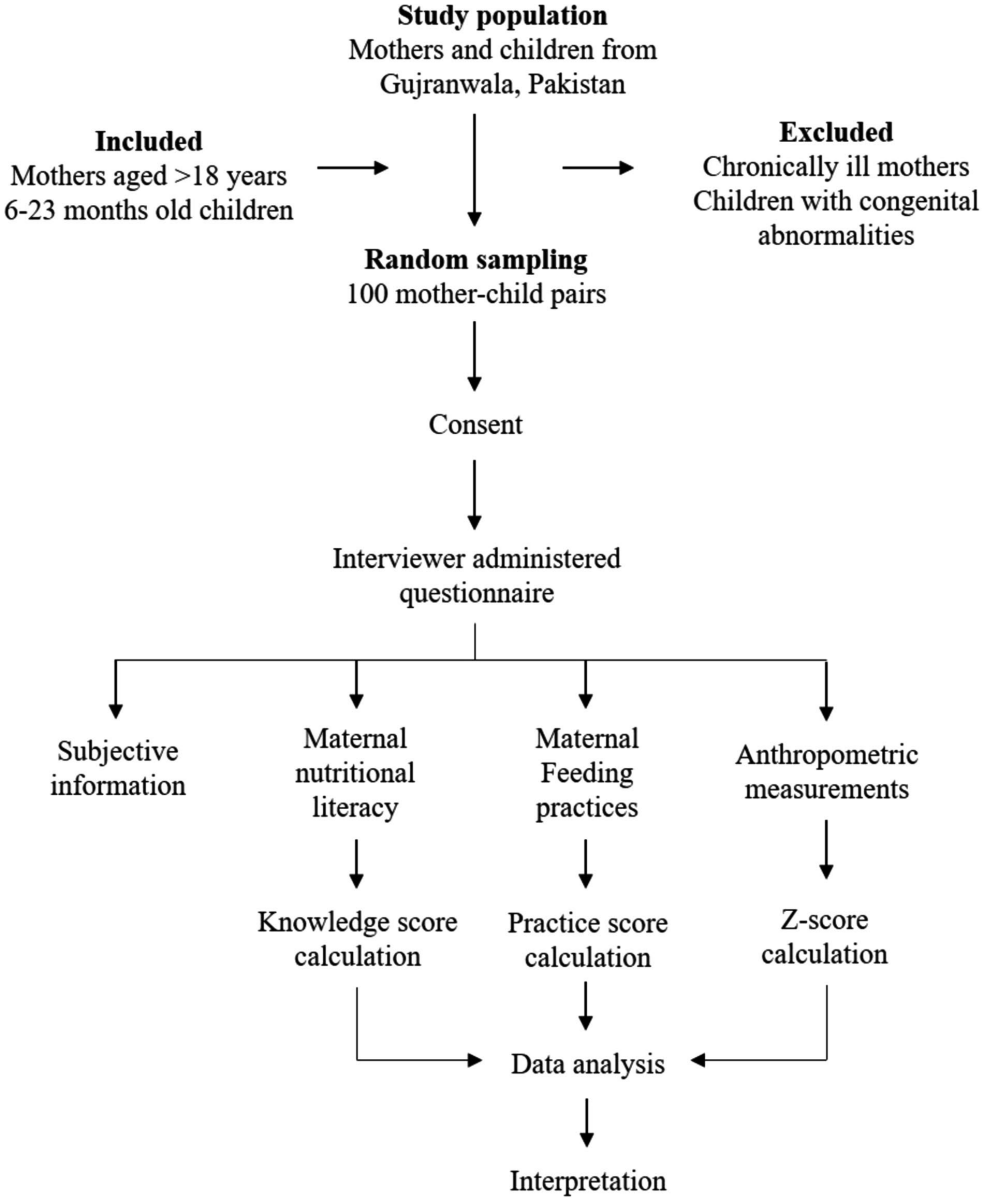
Study plan.

This cross-sectional study based on a household survey was conducted in district Gujranwala, Pakistan to assess the impact of maternal nutritional literacy and feeding practices on child growth outcomes. Hundred mother–child pairs were included in this study from district Gujranwala. The study sample that meets the specific selection criteria was selected randomly. Enrolled children were 6–23 months old and mothers were at least 18 years old, having at least one child aged in the proposed age range at the time of the study. All the chronically ill mothers and children having any congenital abnormalities were excluded from the study. The study plan has been described in Figure 1.

### 2.2 Study instrument

A structured interviewer-administered questionnaire was used to collect the data about maternal nutritional literacy (MNL) and feeding practices (MFP) in the study population. The questionnaire was developed considering the Infant and Young Child Feeding indicators, which are: Ever breastfed (EvBF), early initiation of breastfeeding (EIBF), exclusive breastfeeding for the first 2 days after birth (EBF2D), exclusive breastfeeding for the first 6 months of life (EBF), mixed milk feeding under 6 months (MixMF), continued breastfeeding 12–23 months (CBF), the introduction of solid, semi-solid or soft foods 6–8 months (ISSSF), minimum dietary diversity 6–23 months (MDD), minimum meal frequency 6–23 months (MMF), minimum milk feeding frequency for non-breastfed children 6–23 months (MMFF), minimum acceptable diet 6–23 months (MAD), egg and/or flesh food consumption 6–23 months (EFF), sweet beverage consumption 6–23 months (SwB), unhealthy food consumption 6–23 months (UFC), zero vegetable or fruit consumption 6–23 months (ZVF) and bottle feeding 0–23 months (BoF) (14).

The final version of questionnaire was the combination of WHO model questionnaires and FAO Nutrition-related KAP model questionnaires (14, 15). Furthermore, it was modified such that it best suits the culture and extracts the relevant information regarding the study. In addition to subjective information, the questionnaire had two basic components, i.e., MNL and MFP, followed by the child’s anthropometric measurements.

### 2.3 Ethical consideration

Participants were fully informed about the nature of the study, what their participation entailed, strict confidentiality of their answers and names, and data will be only used for research purposes. They were told they are not obliged to answer any question they do not want to, and they may stop the interview at any time. They then signed the written consent form provided in Urdu and English as per their convenience.

### 2.4 Data collection

Subjective information, maternal knowledge, and practices-related data were collected via a face-to-face interview in a household survey, followed by anthropometric measurements of IYC.

#### 2.4.1 Sociodemographic information

Sociodemographic information included the mother’s name, age, working status, education, family structure, socioeconomic status, child’s name, gender, date of birth, type of birth (vaginal birth or caesarian section), and term of birth (pre-term, term or post-term).

#### 2.4.2 Maternal nutritional literacy

The MNL was assessed by asking 19 questions probing the mothers’ knowledge about the importance of colostrum, appropriate timing for initiation of breastfeeding, duration of exclusive breastfeeding, and timely initiation of complementary feeding along with meal frequency and diet diversification. The responses were preliminarily categorized as “knows” and “do not know.” Then, one mark was given for each correct response. Based on the total number of correct responses, a knowledge score (KS) was given to each respondent. The mean KS of the population and KS for each question were calculated. The knowledge scoring criteria was based on cumulative score which is as follows: poor (<40%), below average (40–59%), average (60–79%), good (80–99%), and excellent (100%).

#### 2.4.3 Maternal feeding practices

Assessment of breastfeeding and complementary feeding practices was done according to the IYCF indicators mentioned by WHO (14). Mothers were questioned about the time of breastfeeding initiation, the age of introducing complementary foods, the child’s usual dietary pattern, type and frequency of milk, meals, and snacks, consumption of unhealthy foods and beverages, etc. Each individual was given one mark for each right practice as per WHO recommendations. Based on the total number of recommended practices, a practice score (PS) was given to each respondent, and the mean PS of the population was computed. The practice scoring criteria was based on cumulative score which is as follows: poor (<40%), below average (40–59%), average (60–79%), good (80–99%), and excellent (100%).

### 2.5 Anthropometric measurements

Anthropometric measurements, i.e., length, weight, head circumference, mid-upper arm circumference, and skinfold thicknesses of the children were assessed by following the standard methods.

#### 2.5.1 Length

The child was correctly aligned on a flat surface with the head in the Frankfort horizontal plane, feet together with toes pointing upwards, and putting slight pressure on the knees. The recumbent length was measured using non-stretchable, non-flexible measuring tape on a metric scale to the nearest 0.1 cm (16).

#### 2.5.2 Weight

Weight was measured by using the digital scale. With minimal clothing and shoes off, the child was laid on the middle of the baby scale. If the child was stable enough to hold the upright position, measurement was taken by making the child stand straight without holding any support such that the weight is uniformly distributed on both feet. Weight was recorded to the nearest 100 g (16).

#### 2.5.3 Head circumference (HC)

Head circumference was measured using a non-stretchable, flexible measuring tape. Measurement was taken by removing the child’s cap or extra hair ornaments, wrapping the tape around the child’s head at the maximal circumference such that it lies around the skull’s frontal bones, above the eyebrows and ears, at a right angle to the long axis of the face and over the occipital prominence at the back of the head. Measurement was recorded on the metric scale to the nearest 0.1 cm (17).

#### 2.5.4 Mid-upper arm circumference (MUAC)

Mid upper arm circumference was assessed by a flexible non-stretchable measuring tape. First of all, the upper arm length was measured by bending the child’s arm at the right angle at the elbow, marking two points, i.e., acromion and olecranon process. At the midpoint of the upper arm, the measuring tape was wrapped vertically and MUAC was recorded to the nearest 0.1 cm (17).

### 2.6 Data analysis

The anthropometric data were analyzed by WHO Antro (version 3.2.2) software to generate WHO child growth standards’ *z*-scores, i.e., length for age (LAZ), weight for age (WAZ), weight for length (WLZ), BMI for age (BMZ), MUAC for age (MUACZ) and HC for age (HCZ). That was used to calculate the prevalence of underweight (WAZ < −2 SD), stunting (LAZ < −2 SD), wasting (WLZ < −2 SD) and overweight (WLZ >+2 SD) in the study population. Additionally, the data were cleaned, and outliers were removed. The *Z*-scores which were outside the WHO flags: WLZ −5 to 5; LAZ −6 to 6; and WAZ −6 to 5 were excluded from the data set. The obtained data were subjected to Statistical Package for Social Sciences (SPSS) for further analysis. Multinomial regression analysis was applied to investigate the association of MNL and MFP with growth outcomes. Intergroup associations were checked by the Chi-square test (18).

## 3 Results

### 3.1 Characteristics of study participants

In the present research, all the study participants were urban residents and most (69%) belonged to middle-income families. An extended family system was found in 86% of families. The mean ages of mothers and children were 28.46 ± 3.836 years and15.10 ± 5.923 months, respectively. More than two-thirds (69%) of mothers in the study population were within the age group of 25–30 years. All the mothers were Muslims and educated, and most (57%) had higher education. The majority (92%) of mothers were housewives. Regarding the sex of IYC, the study comprised 51% males and 49% females. Most (90%) were term infants and Cesarean section was the most common mode of delivery (59%) (Table 1).

**Table 1:** Characteristics of study participants.

		%
Household characteristics		
Socioeconomic status	Low	20
	Middle	69
	High	11
Family structure	Single parent	0
	Nuclear	14
	Extended	86
Residence	Rural	0
	Urban	100
Maternal characteristics		
Age	19–24 years	10
	25–30 years	69
	37–39 years	17
	37–42 years	4
Marital status	Married	100
	Divorced	0
	Widowed	0
Working status	Professionals	8
	Housewives	92
Education	Primary	4
	Middle	5
	Secondary	21
	Higher secondary	13
	Graduation	32
	Post-graduation or above	25
Parity	1 child	34
	2–4 children	64
	5 or more children	2
Child characteristics (*n* = 100)		
Age	6–11 months	34
	12–23 months	66
Gender	Male	51
	Female	49
Mode of delivery	Vaginal	41
	Cesarean section	59
Term of birth	Pre-term	10
	Term	90
	Post-term	0

### 3.2 Maternal nutritional literacy in the study population

The mean KS of the respondents was 14.29 ± 2.176 out of a total of 19. The minimum and maximum KS were recorded as 9 and 19, respectively. Mothers’ knowledge was high in terms of the first food of new-born (93%), understanding about EBF (90%), need for medicines or oral supplements under 6 months (90%), the importance of breastmilk (83%), benefits of EBF (96%), the superiority of breastmilk over formulas (99%), need of CF (100%), foods that can be added to porridge (96%) and MDD (95%). Knowledge gaps were observed in porridge consistency (26%), MMF (42%), the timing for breastfeeding initiation (53%), and water supplementation under 6 months (55%). In total, more than half of the mothers (57%) were found to have average knowledge of IYCF components, 29% had good knowledge, 13% had below-average knowledge and only 1% had excellent knowledge (Table 2).

**Table 2:** Maternal nutritional literacy in the study population.

Correctly answered knowledge-related items	Correct (%)	Incorrect (%)	Mean score
Breastmilk should be the first food of new-born	93	7	0.93
Colostrum is the yellowish secretion after delivery	72	28	0.72
Colostrum is beneficial for the baby	64	36	0.64
Breastfeeding should be initiated within 1 h of birth	53	47	0.53
An infant should be provided only breastmilk, and no other liquid or food in the first 6 months	86	14	0.86
Water supplementation is not needed in the first 6 months	55	45	0.55
Medicines/oral supplements can be provided to an infant when indicated	92	8	0.92
The optimal duration of exclusive breastfeeding (EBF) is 6 months	73	27	0.73
EBF is preferred as breastmilk is nutritionally adequate and/or the infant cannot digest anything other than breastmilk in the first 6 months	83	17	0.83
An infant should be breastfed on demand	66	34	0.64
Breastmilk is better at fulfilling the infant’s nutritional requirements than infant formula	99	1	0.99
Breastfeeding should be continued for 2 years or beyond	79	21	0.79
Six months is the right age for introducing complementary foods	61	39	0.61
Complementary foods are needed as breast milk alone is not sufficient after a certain age	100	0	1
Thick porridge should be given as its more nutritious	26	74	0.26
At least five food groups per day must be consumed by children	95	5	0.95
Children must consume multiple meals/snacks depending on their breastfeeding status	27	73	0.27

### 3.3 Maternal feeding practices in the study population

The mean practice score of 6.18 ± 2.13 out of a total of 16. About 18% of infants were never breastfed in their early years of life and 12% of mothers initiated breastfeeding within 1 h of birth. Even though 32% of newborns were fed exclusively in the first 2 days of life, only 7% were exclusively breastfed by the end of the sixth month. In total, 95% of infants received animal milk and/or formula in addition to breast milk before the infant reaches 6 months. Among children aged 12–23 months (*n* = 66), only 27% continued to be breastfed beyond 1 year. Complementary foods were timely introduced to 86% of infants. 53% of infants did not receive minimum meal frequency and 7.45% failed to receive minimum milk feeding frequency. The IYC that did not fulfill the minimum dietary diversity and minimum acceptable diet criteria were 66 and 76%, respectively. Egg and flesh foods were consumed by 71% of IYC. Additionally, consumption of unhealthy foods, sugar-sweetened beverages, and zero consumption of fruits and vegetables were practiced by 71, 23, and 27% of IYC, respectively. The proportion that used feeding bottles was 91%. So, maternal feeding practices were not satisfactory as more than half (58%) of mothers were found to have poor feeding practices, 35% had below average practices and 7% had average practices and none fell in the good or excellent category (Table 3).

**Table 3:** Maternal feeding practices in the study population.

IYCF indicators	No (%)	Yes (%)	Total
Ever breastfed (EvBF)	18	82	100
Early initiation of breastfeeding (EIBF)	88	12	100
Exclusive breastfeeding for the first 2 days (EBF2D)	68	32	100
Exclusive breastfeeding for the first 6 months of life (EBF)	93	7	100
Mixed milk feeding under 6 months (MixMF)	95	5	100
Continued breastfeeding (CBF)	73	27	66
Introduction of solid, semi-solid, or soft foods (ISSSF)	14	86	21
Minimum dietary diversity (MDD)	66	34	100
Minimum meal frequency (MMF)	53	47	100
Minimum milk feeding frequency (MMFF)	7	93	67
Minimum acceptable diet (MAD)	76	24	100
Egg and/or flesh food consumption (EFF)	49	51	100
Sweet beverage consumption (SwB)	53	47	100
Unhealthy food consumption (UFC)	29	71	100
Zero vegetable or fruit consumption (ZVF)	27	73	100
Bottle feeding (BoF)	9	91	100

### 3.4 Growth outcomes of children by age

**Figure 2:**
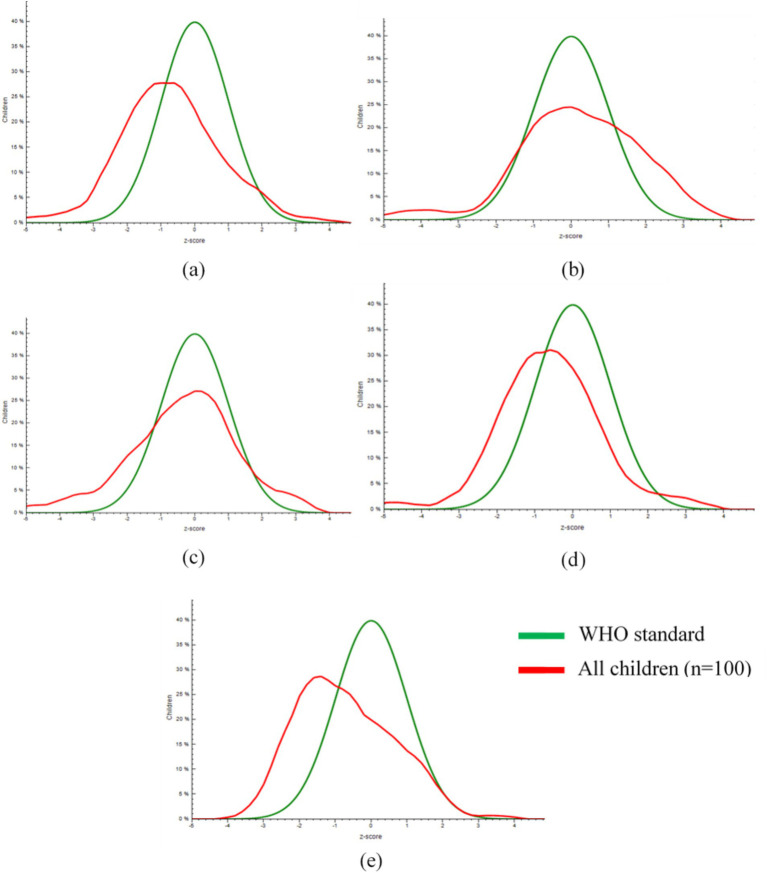
Distribution of length-for-age *z*-scores **(a)**, weight-for-length *z*-scores **(b)**, weight-for-age *z*-scores **(c)**, head circumference-for-age *z*-scores **(d)** and mid-upper arm circumference-for-age *z*-scores **(e)** of IYC in comparison with the reference distribution established by WHO.

The mean LAZ of IYC in the study population was −0.74 ± 1.46 SD. However, Figure 2A demonstrates a left deviation in the entire LAZ distribution of the study population in contrast to the WHO reference distribution. This indicates some degree of growth faltering among all the children let alone those falling below a specific cut-off. The mean WLZ of IYC in the study population was 0.19 ± 1.58 SD. Nevertheless, the disproportionate WLZ of the study population in contrast to the WHO reference distribution is noteworthy. Figure 2B suggests the coexistence of wasting and overweight in the study population. The mean WAZ of IYC in the study population was −0.39 ± 1.6 SD. Figure 2C manifests a left deviation in the WAZ of the study population in contrast to the WHO reference distribution. That suggests the inclination of the study population toward underweight. Likewise, a left deviation in the entire HCZ and MUACZ distribution of the study population has been shown in Figures 2D,E, respectively. Thus, indicating some degree of brain growth deficits and malnutrition among all the children in the study population let alone those falling below a specific cut-off. Prevalence of stunting, wasting, underweight and overweight was 19, 9, 12, and 12%, respectively (Table 4). Of these, 63.1, 33.3, 33.3, and 75%, respectively were 12–23 months old.

**Table 4:** Growth outcomes of children by age (*n* = 100).

Growth outcomes	Child age				Total	
	6–11 months		12–23 months			
	*n*	%	*n*	%	*n*	%
Stunting (LAZ)	7	20.58	12	18.18	19	19
Wasting (WLZ)	6	17.64	3	4.54	9	9
Underweight (WAZ)	8	23.52	4	6.06	12	12
Overweight (WLZ)	3	8.82	9	13.63	12	12

### 3.5 Association between maternal feeding practices and undernutrition

#### 3.5.1 Association between maternal nutritional literacy and stunting

The bivariate association between maternal nutritional literacy (MNL) and stunting signifies that MNL was not significantly associated with stunting at *p* < 0.05 (Table 5). The covariates found to be significantly associated with stunting include the mother’s age and education (Table 6). The potential confounders were adjusted by subjecting the data to a multinomial regression model. The goodness of fit test was not statistically significant (*X*^2^ = 40.006, df = 54, *p* = 0.922), indicating model adequacy. The research findings suggested a non-significant association between MNL and stunting. However, the mother’s education failed to reach significance in multivariate analysis (Table 7).

**Table 5:** Bivariate association of maternal nutritional literacy and maternal feeding practices with growth outcomes of children.

	Maternal nutritional literacy		Maternal feeding practices	
Growth outcomes	*X* ^2^	*p*-value	*X* ^2^	*p*-value
Stunting (LAZ)	2.677	0.848^NS^	2.541	0.637^NS^
Wasting (WLZ)	5.595	0.470^NS^	0.883	0.927^NS^
Underweight (WAZ)	7.525	0.275^NS^	3.336	0.503^NS^

^NSNon-significant.^

C.I. < 0.05.

**Table 6:** Association between factors and growth outcomes.

	Length-for-age *z*-score		Weight-for-length *z*-score		Weight-for-age *z*-score	
Factors	*X* ^2^	*p*-value	*X* ^2^	*p*-value	*X* ^2^	*p*-value
Mother’s age	24.330	0.000*	2.117	0.909^NS^	11.451	0.075^NS^
Mother’s education	20.709	0.023*	8.469	0.583^NS^	15.387	0.119^NS^
Mother’s working status	0.666	0.717^NS^	2.174	0.337^NS^	0.759	0.684^NS^
Type of family	1.767	0.413^NS^	2.174	0.337^NS^	2.824	0.244^NS^
Socioeconomic status	4.137	0.388^NS^	7.455	0.114^NS^	9.237	0.055^NS^
No of children	0.818	0.936^NS^	6.068	0.194^NS^	1.112	0.892^NS^
Child’s age	0.605	0.739^NS^	4.771	0.092^NS^	7.624	0.022*
Child’s gender	1.551	0.460^NS^	1.502	0.472^NS^	1.744	0.418^NS^
Mode of delivery	1.437	0.487^NS^	6.057	0.048*	6.521	0.038^*^
Term of birth	3.440	0.179^NS^	1.818	0.403^NS^	1.528	0.466^NS^

*Significant.

^NSNon-significant.^

C.I. < 0.05.

**Table 7:** Multivariate association of maternal nutritional literacy and maternal feeding practices with growth outcomes of children.

			Maternal Nutritional Literacy			Maternal Feeding Practices		
			Coeff. (*β*)	*p*-value	Exp(B)	Coeff. (*β*)	*p*-value	Exp(B)
Stunting^a^	Stunted	Intercept	−16.830	0.000*		−17.947	0.000*	
		Knowledge and practice scores	−0.468	0.298^NS^	0.626	−0.421	0.370^NS^	
		Mother’s age (19–24 years)	16.549	0.000*	1.538	16.783	0.000*	1.944
		Mother’s age (25–30 years)	16.672	0.000*	1.739	16.770	0.000*	1.919
		Mother’s age (31–36 years)	17.185		2.905	17.273		3.173
		Mother’s age (37–42 years)	0			0		
		Mother’s education (primary)	0.296	0.822^NS^	1.344	0.452	0.726^NS^	1.572
		Mother’s education (middle)	1.904	0.091^NS^	6.713	2.173	0.049*	8.788
		Mother’s education (secondary)	−0.282	0.753^NS^	0.754	−0.077	0.930^NS^	0.926
		Mother’s education (higher secondary)	0.027	0.978^NS^	1.028	0.358	0.716^NS^	1.431
		Mother’s education (graduation)	0.158	0.829^NS^	1.172	0.322	0.659^NS^	1.380
		Mother’s education (post-graduation)	0			0		
	Tall	Intercept	−12.611	0.934^NS^		−11.693	0.937^NS^	
		Knowledge and practice scores	0.476	0.722^NS^	1.610	0.595	0.673^NS^	1.814
		Mother’s age (19–24 years)	−13.056	0.968^NS^	0.000	−14.011	0.964^NS^	
		Mother’s age (25–30 years)	−3.462	0.047*	0.031	−3.806	0.053^NS^	0.022
		Mother’s age (31–36 years)	−1.995	0.279^NS^	0.136	−2.184	0.228^NS^	0.113
		Mother’s age (37–42 years)	0			0		
		Mother’s education (primary)	0.143	1.000^NS^	1.154	0.254	1.000^NS^	1.289
		Mother’s education (middle)	1.529	0.998^NS^	4.614	1.516	0.998^NS^	4.552
		Mother’s education (secondary)	0.398	0.999^NS^	1.489	0.314	0.999^NS^	1.369
		Mother’s education (higher secondary)	12.508	0.935^NS^	2,704	12.410	0.933^NS^	2,452
		Mother’s education (graduation)	10.573	0.945^NS^	3,905	10.439	0.944^NS^	3,417
		Mother’s education (Post-graduation)	0			0		
Wasting^a^	Wasted	Intercept	0.546	0.775^NS^		−2.903	0.022*	
		Knowledge and practice scores	−1.300	0.052^NS^	0.273	−0.189	0.781^NS^	0.828
		Type of birth (vaginal)	1.903	0.029*	6.706	1.660	0.051^NS^	5.257
		Type of birth (C-section)	0			0		
	Obese	Intercept	−3.095	0.069^NS^		−1.864	0.041*	
		Knowledge and practice scores	0.392	0.430^NS^	1.480	0.032	0.951^NS^	1.032
		Type of birth (vaginal)	−0.530	0.460^NS^	0.589	−0.514	0.482^NS^	0.598
		Type of birth (C-section)	0			0		
Underweight^a^	Underweight	Intercept	0.213	0.775^NS^		−3.116	0.024*	
		Knowledge and practice scores	−1.204	0.052^NS^	0.300	−0.230	0.743^NS^	0.794
		Child’s age (6–11 months)	1.058	0.029*	2.880	1.363	0.055^NS^	3.908
		Child’s age (12–23 months)	0			0		
		Type of birth (vaginal)	1.732	0.024*	5.654	1.551	0.039*	4.714
		Type of birth (C-section)	0			0		
	Overweight	Intercept	−3.171	0.069^NS^		−1.786	0.104^NS^	
		Knowledge and practice scores	0.330	0.430^NS^	1.391	−0.184	0.763^NS^	0.832
		Child’s age (6–11 months)	−1.126	0.460^NS^	0.324	−1.206	0.276^NS^	0.300
		Child’s age (12–23 months)	0			0		
		Type of birth (vaginal)	0.035	0.964^NS^	1.036	0.039	0.960^NS^	1.039
		Type of birth (C-section)	0			0		

*Significant.

^NSNon-significant.^

C.I. < 0.05.

#### 3.5.2 Association between maternal nutritional literacy and wasting

The bivariate association between maternal nutritional literacy (MNL) and wasting signifies that MNL was not significantly associated with wasting at *p* < 0.05 (Table 5). The covariate found to be significantly associated with wasting include mode of delivery (Table 6). A multinomial regression model was applied for adjusting the potential confounders in the model. The goodness of fit test was not statistically significant (*X*^2^ = 4.568, df = 8, *p* = 0.803), indicating model adequacy. Results showed a non-significant association between MNL and wasting. Furthermore, the type of birth-maintained significance in multivariate analysis (Table 7).

#### 3.5.3 Association between maternal nutritional literacy and underweight

The bivariate association between maternal nutritional literacy (MNL) and underweight signifies that MNL was not significantly associated with underweight at *p* < 0.05 (Table 5). The covariates found to be significantly associated with being underweight were the child’s age and mode of delivery (Table 6). The potential confounders in the model were adjusted by using multinomial logistic regression analysis. The goodness of fit test was not statistically significant (*X*^2^ = 16.706, df = 16, *p* = 0.405), indicating model adequacy. The study showed a non-significant association between MNL and underweight. Nevertheless, covariates failed to reach significance in multivariate analysis (Table 7).

### 3.6 Association between maternal feeding practices and undernutrition

#### 3.6.1 Association between maternal feeding practices and stunting

The bivariate association between maternal feeding practices (MFP) and stunting signifies that MFP was not significantly associated with stunting at *p* < 0.05 (Table 5). For adjusting the potential confounders in the model, the multinomial regression analysis was applied. The goodness of fit test was not statistically significant (*X*^2^ = 27.518, df = 44, *p* = 0.975), indicating model adequacy. Results showed a non-significant association between MFP and stunting. However, none of the covariates achieved significance (Table 7).

#### 3.6.2 Association between maternal feeding practices and wasting

The bivariate association between maternal feeding practices (MFP) and wasting signifies that MFP was not significantly associated with wasting at *p* < 0.05 (Table 5). The potential confounders in the model were adjusted by using the multinomial logistic regression model. The goodness of fit test was not statistically significant (*X*^2^ = 0.704, df = 6, *p* = 0.994), indicating model adequacy. After adjusting the potential confounders, the study showed a non-significant association between MFP and wasting (Table 7).

#### 3.6.3 Association between maternal feeding practices and underweight

The bivariate association between maternal feeding practices (MFP) and underweight signifies that MFP was not significantly associated with underweight at *p* < 0.05 (Table 5). The potential confounders in the model were adjusted by using a multinomial logistic regression model. The goodness of fit test was not statistically significant (*X*^2^ = 8.768, df = 14, *p* = 0.846), indicating model adequacy. The final model showed a non-significant association between MFP and underweight (Table 7).

## 4 Discussion

### 4.1 Maternal nutritional literacy in the study population

This survey showed that maternal knowledge about colostrum was generally average. The majority of the mothers (93%) knew that breastmilk should be the first food of new-born. About 72% of mothers knew what colostrum is and described it as watery yellowish secretion after delivery. Additionally, 64% of the respondents reported colostrum to be good for the baby. The percentage of mothers knowing the importance of colostrum was comparatively lower than a study in Saudi Arabia (89.3%) but higher than the trends observed in Ethiopia, i.e., 60.2% (11, 19). The differences in results can be due to sociodemographic variations. More than half of mothers (53%) responded that the baby should be breastfed immediately after birth. The observed trends were lower than that reported by two other studies, i.e., 73.2 and 86.3% (19, 20). The plausible explanation for differences in results could be differences in the level of education, traditional beliefs about colostrum being harmful to babies, and practices of pre-lacteal feeding. The mothers’ understanding of EBF was generally fair while recognizing several noteworthy inadequacies. The reason is that 43% of mothers exempted water from the category of fluids, and others considered the prescribed medication/oral supplements not to be provided to the infant when needed. The results reported by Yeshambel Wassie et al. (20) were comparable to the findings of this research project (43%).

Almost 73% of mothers knew the optimal duration of EBF, 8% did not know the answer and others provided varying durations contradicting the guidelines. The results achieved in the current research are comparable to Arzu et al. (12) as 70.5% of mothers were aware of the duration of EBF. 40% of mothers in the current research considered the breastmilk complete in terms of providing all the nutrients with an infant needs in the first 6 months. Moreover, 4% responded that babies cannot digest anything other than breastmilk before 6 months and 39% agreed with both statements. All responses were considered correct so in total, 83% of mothers were knowledgeable. The obtained results were almost similar to Al Ketbi et al. (21) as 81.2% of mothers were aware that breastmilk is sufficient for fulling dietary requirements in the first 6 months of life. Regarding frequency, 66% of mothers knew that the child should be fed on demand, while 34% were in favor of scheduled feeding. The results of this research were comparable to Iacovou and Sevilla (22) as 69.8% of mothers preferred feeding on demand over scheduled feeding. 79% of mothers were aware that breastfeeding should be continued for 2 years or beyond. The study findings were comparable to previous studies from India and Nepal, where the proportion of mothers knowing the appropriate duration was 68 and 75%, respectively (23, 24). The current study demonstrated that in total, only 24% of mothers came up with recommended meal frequency and 4% had no idea about meal frequency. The proportion of mothers who knew the right meal frequency for IYC was far less than in another study conducted in Lahore, Pakistan, where 80% of mothers were knowledgeable regarding meal frequency (13). Overall, more than half of the mothers (57%) were found to have average knowledge of IYCF components, 29% had good knowledge, 13% had below-average knowledge and only 1% had excellent knowledge. Results were close to another study as 24% of respondents had good knowledge (>70% KS) regarding complementary feeding whereas satisfactory and poor knowledge were found in 48 and 28% of respondents, respectively (13).

### 4.2 Maternal feeding practices in the study population

Although breastfeeding was universal (82%) in the study area, only 12% of mothers initiated breastfeeding within the first hour of birth. A delay in breastfeeding initiation could be explained by the fact that 59% of newborns in the study population were delivered via cesarean section (CS). CS may have detrimental effects on early breastfeeding behaviors and long-term breastfeeding outcomes (25). Lack of knowledge, traditional beliefs, pre-lacteal feeding, elderly advice, cesarean section delivery, no milk secretion, and ill health of either mother or baby are some of the known barriers to EIBF in South Asia (26). The results from this research were comparable to another study from Pakistan that reported EIBF in only 14% of newborns (27). The slight difference might be due to the difference in the sample sizes. In the current study, 68% of newborns were not exclusively breastfed for the first 2 days after birth. Breastfeeding during the first few days is often overlooked and feeding something other than breastmilk is a common practice. Studies suggest that EBF has a significant effect on preventing infant morbidity and mortality. Rather, mixed milk feeding during the first 3 days is a significant predictor of early cessation of EBF (28). Even though 32% of newborns were fed exclusively in the first 2 days of life, only 7% were exclusively breastfed by the end of the sixth month. The reasons are the early introduction of mixed milk feeding, water supplementation under 6 months, and the early introduction of complementary foods. The obtained results were farther from that reported by NNS, i.e., 48% EBF rates (2).

In the study population, 95% of infants received MixMF under 6 months. A systematic review highlighted the highest prevalence of MixMF in Asia with sustained high rates across 6 months of age. The identified major drivers for preferring MixMF over EBF were insecurity of mothers about satisfying their infants’ needs, breastfeeding in public, avoiding exclusive formula feeding, having social flexibility, perceived advantages, cultural beliefs, maternal health issues, etc. (29). Out of 66 children aged 12–23 months, only 18 continued to be breastfed beyond 1 year. That accounts for 27.27% of IYC of that age and only 21.66% of mothers intending to continue breastfeeding for up to 2 years. The obtained results were very close to that reported by Al Ketbi et al. (21). Out of 21 infants aged 6–8 months, 18 infants which account for 86% consumed any solid, semi-solid or soft food (SSSF) on the day before the interview while SSSF was not provided to 14% of infants in the study population. The proportion introduced complementary foods at the right age was comparable to that reported by Hasnain et al. (13). 34% of IYC in this study consumed a diet comprising 5 or more food groups. The research findings were in accordance with Tegegne et al. (30). 47% of IYC received MMF. This figure was very close to that reported in India, i.e., 41.5% (31). The slight difference might be due to the cultural and religious differences among the study population. Moreover, 89% of IYC who participated in the study belonged to low and middle-income families as household resources are an important determinant of the quantity and quality of food for children. In the study population, only 24% of IYC consumed MAD while 76% failed to meet the MAD criteria. Tegegne et al. (30) reported similar findings, i.e., MAD was provided to only 26.8% of IYC in his study. Overall, more than half, i.e., 58% of mothers were found to have poor feeding practices, 35% had below average practices and 7% had average practices and none fell in the good or excellent category. Irrespective of maternal nutritional literacy (57%), feeding practices were not satisfactory as practices are largely driven by cultural beliefs and preferences.

### 4.3 Association between maternal nutritional literacy and undernutrition

The research findings suggested a non-significant association between MNL and stunting with some anomalies. For IYC of mothers aged between 19 to 24 years, the odds of being stunted vs. normal are 1.538 times higher [Exp(b) = 1.538] compared to those who aged higher than that. Having a mothers aged between 25 and 30 years, the odds of being stunted increases by a factor of 1.739 [Exp(b) = 1.739] compared to the reference group. For IYC of mothers aged between 25 and 30 years, the odds of being tall vs. normal ones are 0.031 times lower [Exp(b) = 0.031] compared to those who aged other than that. This might be due to the fact that MNL and MFP were not in concert with each other in the study population. About 57% of mothers had average nutritional knowledge, whereas 58% had poor feeding practices. As it is evident that knowledge did not seem to translate into practices that’s why results showed undesirable nutritional outcomes irrespective of the knowledge score. The results of this study are in accordance with Webb and Block (32) who found that nutritional knowledge was not associated with LAZ.

With only one exception, a non-significant association between MNL and wasting was observed in this study. The IYC who had vaginal birth, the odds of being wasted vs. normal ones are 6.706 times higher [Exp(b) = 6.706] compared to those who were delivered by C-Section. Since the MFP did not yield comparable results to MNL that might be the reason behind the non-significant findings. Moreover, MNL is just one of the factors influencing growth outcomes, others being the wealth index of the family, the mother’s education, access to antenatal care services, the child’s age, breastfeeding status of the child, etc. (33). The results of this research were close to that reported by Mueni (34).

The study showed a non-significant association between MNL and underweight with only one exception. The IYC who had vaginal birth, the odds of being underweight vs. normal ones are 5.654 times higher [Exp(b) = 5.654] compared to those who were delivered by C-Section. This might be due to multiple reasons that are unsatisfactory knowledge among mothers about appropriate feeding practices, poor implementation of existing knowledge, inclination toward cultural practices, etc. Moreover, insignificant differences among mothers in terms of MNL and MFP led to disproportionate WAZ in the study population, resultantly failure to reach significance. The results of this research were comparable to Mueni (34) that reported statistically non-significant relationship between underweight and most of knowledge indicators, i.e., 16 out of 20 indicators.

### 4.4 Association between maternal feeding practices and undernutrition

Results showed a non-significant association between MFP and stunting with certain exceptions. For IYC of mothers aged between 19 and 24 years, the odds of being stunted vs. normal are 1.944 times higher [Exp(b) = 1.944] compared to those who aged higher than that. Having a mothers aged between 25 and 30 years, the odds of being stunted increases by a factor of 1.919 [Exp(b) = 1.919] compared to the reference group. The IYC whose mother had middle school education, the odds of being stunted vs. normal ones are 8.786 times higher [Exp(b) = 8.786] compared to those having higher education. This might be due to the small sample size, recall biases, forgetfulness about the BF practices in the first 6 months, and limitation of IYCF indicators to rely on 24 h reference period for CF practices assessment. The 24 h recall reflects the recent diet, in contrast, stunting indicates an individual’s long-term nutritional status. In consonance with the finding of this research, Meshram et al. (35) found a non-significant association between BF practices and LAZ. Anin et al. (36) in a study conducted in Northern Ghana reported that none of the individual CF indicators was associated with LAZ, except timely introduction to complementary feeding. Likewise, some other studies in southern Ethiopia and Cambodia showed a weak association between stunting and IYCF indicators (37, 38).

The study showed a non-significant association between MFP and wasting. The lack of significance may be the result of insignificant differences in feeding practices across the study population and the inclination of the whole population toward the poor, below average, and average practices. Most importantly, feeding practices are only one aspect of determining a child’s growth. Studies suggest that other factors include the household’s position in the wealth index, geographic distribution, maternal BMI, height and age, access to antenatal visits, type of birth facility, child’s gender, and birth order (39, 40). The finding of this research is in accordance with studies conducted in India and Northern Ghana that showed a non-significant association between IYCF indicators and wasting (35, 36).

A non-significant association between MFP and underweight was observed in this research with only one exception. The IYC who had vaginal birth, the odds of being underweight vs. normal ones are 4.714 times higher [Exp(b) = 4.714] compared to those who were delivered by C-Section. The apparent lack of association may be due to the fact there was very little variation in the study population with respect to these indicators. Moreover, feeding indicators may not be sensitive to chronic undernutrition because their assessment is based on 24 h recall that may not give the usual dietary intake. The results achieved in current research are comparable to Menon et al. (41) as they described that none of the IYCF indicators was associated with underweight except ISSSF and MDD.

## 5 Strengths and weaknesses of the study

High quality primary data was obtained involving standard interviewer training and anthropometric measurements training. However, being a cross-sectional study, causality cannot be inferred from the results. Due to the limited availability of similar studies conducted in Gujranwala, some comparisons of the IYCF indicators were not ideal. At the time of study, new IYCF indicators were introduced by WHO so inadequate studies were available for making appropriate comparisons. Even though 24 h. dietary recall is widely accepted in nutritional epidemiological studies, still this method poses certain challenges like recall bias, the desire to give socially desirable responses and forgetfulness thus have affected some of the findings. The sample size was small so the results could not be generalized to whole population.

## 6 Recommended public health interventions

The interventions that address dietary diversity like the community-based promotion of improved IYCF practices, and unconditional cash transfers should be pursued, together with reduction in the burden of infectious diseases. Ensuring the surplus availability of locally cultivated fruits and vegetables at low price to improve the quality of child food intake. The local, national, and international stakeholders should engage in improve children’s diets in LMICs during the first 2 years of life, as a priority area. The government needs to ensure that its food and nutrition security policies are locally relevant in order to promote good breastfeeding and complimentary feeding practices. Nutrition-sensitive interventions, such as vaccination programs and child immunization, promotion of appropriate water, sanitation and hygiene (WASH) practices, improving agriculture and food security in households, should be implemented and scaled up. Behavior change education (BCE) targeted at improving nutrition-specific IYCF practices, should be continually carried out. Additionally, nutrition-specific interventions such as the production and distribution of special nutrient-rich foods from local food ingredients could help address acute undernutrition.

## 7 Conclusion

The suboptimal feeding practices were widespread despite the average nutritional knowledge among mothers in the district Gujranwala, Pakistan. MNL and MFP had a non-significant association with growth indicators. There is a dire need to not only create nutritional awareness among mothers but also provide a support system to help them implement standard feeding practices. Furthermore, societal barriers to implementing optimal feeding practices need to be addressed. Further studies are required in this context. The sample size can be increased to have more accurate judgments and subjects should be selected from varied regions to get results that can be generalized for the population.

## Data Availability

The raw data supporting the conclusions of this article will be made available by the authors, without undue reservation.

## References

[1] UNICEF. Malnutrition prevalence remains alarming: stunting is declining too slowly while wasting still impacts the lives of far too many young children [internet]. (2020) [cited 2020 Nov 16]. Available at: https://data.unicef.org/topic/nutrition/malnutrition (Accessed November 16, 2020).

[2] GOP. Key findings of National Nutrition Survey Pakistan 2018 [internet]. Islamabad, Pakistan; (2019). Available at: https://www.unicef.org/pakistan/media/1951/file/Final.Key.Findings.Report.2019.pdf (Accessed November 16, 2020).

[3] Bhutta ZA Das JK Rizvi A Gaffey MF Walker N Horton S. Evidence-based interventions for improvement of maternal and child nutrition: what can be done and at what cost? Lancet. (2013) 382:452–77. doi: 10.1016/S0140-6736(13)60996-4, PMID: 23746776

[4] WorldBank. The World Bank and nutrition [internet]. (2019) [cited 2021 Feb 18]. Available at: https://www.worldbank.org/en/topic/nutrition/overview/ (Accessed February 18, 2021).

[5] Kinyoki DK Osgood-Zimmerman AE Pickering BV Schaeffer LE Marczak LB Lazzar-Atwood A. Mapping child growth failure across low- and middle-income countries. Nature. (2020) 577:231–4. doi: 10.1038/s41586-019-1878-8, PMID: 31915393 PMC7015855

[6] Martorell R. Improved nutrition in the first 1000 days and adult human capital and health. Am J Hum Biol. (2017) 29:1–24. doi: 10.1002/ajhb.22952, PMID: 28117514 PMC5761352

[7] UNICEF, WHO. Capture the moment – Early initiation of breastfeeding: the best start for every newborn. New York, USA: UNICEF (2018).

[8] UNICEF. Adopting optimal feeding practices is fundamental to a child’s survival, growth and development, but too few children benefit [internet]. (2019) [cited 2020 Nov 16]. Available at: https://data.unicef.org/topic/nutrition/infant-and-young-child-feeding/ (Accessed November 16, 2020).

[9] Prasetyo YB Permatasari P Susanti HD. The effect of mothers’ nutritional education and knowledge on children’s nutritional status: a systematic review. Int J Child Care Educ Policy. (2023) 17, 11–26. doi: 10.1186/s40723-023-00114-7, PMID: 39680432

[10] Agize A Jara D Dejenu G. Level of knowledge and practice of mothers on minimum dietary diversity practices and associated factors for 6–23-month-old children in Adea Woreda, Oromia. Ethiopia Biomed Res Int. (2017) 2017:1–9. doi: 10.1155/2017/7204562, PMID: 28497063 PMC5405353

[11] Al-Binali AM. Breastfeeding knowledge, attitude and practice among school teachers in Abha female educational district, southwestern Saudi Arabia. Int Breastfeed J. (2012) 7:10–5. doi: 10.1186/1746-4358-7-10 22894174 PMC3579703

[12] Arzu T Sujan AK Juliana FM Hossain S. Study of IYCF indicators on practices and knowledge of mothers in rural areas. Am J Public Heal Res. (2018) 6:130–3. doi: 10.12691/ajphr-6-3-1

[13] Hasnain S Majrooh MA Anjum R. Knowledge and practices of mothers for complementary feeding in babies visiting pediatrics outpatient Department of Jinnah Hospital, Lahore. Biomedica. (2013) 29:221–30.

[14] WHO, UNICEF. Indicators for assessing infant and young child feeding practices: definitions and measurement methods [internet]. WHA55 A55/, World Health Organization and the United Nations Children’s fund (UNICEF). Geneva; (2021). Available at: http://apps.who.int/iris/bitstream/handle/10665/44306/9789241599290_eng.pdf?sequence=1%0Ahttp://whqlibdoc.who.int/publications/2008/9789241596664_eng.pdf%5Cnhttp://www.unicef.org/programme/breastfeeding/innocenti.htm%5Cnhttp://innocenti15.net/declaration (Accessed February, 20 2020).

[15] FAO. Guidelines for assessing nutrition-related knowledge, attitudes and practices – KAP manual. Rome: Food and Agriculture Organization of the United Nations. (2014). 1–188.

[16] WHO. Training course on child growth assessment. Geneva: WHO (2008).

[17] CDC. National Health and nutrition examination survey (NHANES) anthropometry procedures manual. Atlanta, Georgia, United States: US Department of Health and Human Services (2020).

[18] Montgomery DC. Design and analysis of experiments. 7th ed. Hoboken, NJ, USA: John Wiley and Sons Inc. (2017).

[19] Tadele N Habta F Akmel D Deges E. Knowledge, attitude and practice towards exclusive breastfeeding among lactating mothers in Mizan Aman town, southwestern Ethiopia: descriptive cross-sectional study. Int Breastfeed J. (2016) 11:3–9. doi: 10.1186/s13006-016-0062-0, PMID: 26925156 PMC4769508

[20] Yeshambel Wassie A Atnafu Gebeyehu N Abebe GK. Knowledge, attitude, and associated factors towards colostrum feeding among antenatal care attendant mothers in Gununo health Centre, Wolaita zone, Ethiopia 2019: cross-sectional study. Int J Pediatr. (2020) 2020:1–10. doi: 10.1155/2020/3453502, PMID: 32099549 PMC6996677

[21] Al Ketbi MI Al Noman S Al Ali A Darwish E Al Fahim M Rajah J. Knowledge, attitudes, and practices of breastfeeding among women visiting primary healthcare clinics on the island of Abu Dhabi, United Arab Emirates. Int Breastfeed J. (2018) 13:26–39. doi: 10.1186/s13006-018-0165-x, PMID: 29988693 PMC6029179

[22] Iacovou M Sevilla A. Infant feeding: the effects of scheduled vs. on-demand feeding on mothers’ wellbeing and children’s cognitive development. Eur J Pub Health. (2013) 23:13–9. doi: 10.1093/eurpub/cks012, PMID: 22420982 PMC3553587

[23] Chaudhary R Shah T Raja S. Knowledge and practice of mothers regarding breast feeding: a hospital based study. Heal Renaiss. (2011) 9:194–200. doi: 10.3126/hren.v9i3.5590, PMID: 37609976

[24] Vijayalakshmi P Susheela T Mythili D. Knowledge, attitudes, and breast feeding practices of postnatal mothers: a cross sectional survey. Int J Health Sci. (2015) 9:364–74. doi: 10.12816/0031226, PMID: 26715916 PMC4682591

[25] Zhang F Cheng J Yan S Wu H Bai T. Early feeding behaviors and breastfeeding outcomes after cesarean section. Breastfeed Med. (2019) 14:325–33. doi: 10.1089/bfm.2018.0150, PMID: 30864825

[26] Sharma IK Byrne A. Early initiation of breastfeeding: a systematic literature review of factors and barriers in South Asia. Int Breastfeed J. (2016) 11:17–28. doi: 10.1186/s13006-016-0076-7, PMID: 27330542 PMC4912741

[27] Zakar R Zakar MZ Zaheer L Fischer F. Exploring parental perceptions and knowledge regarding breastfeeding practices in Rajanpur, Punjab Province, Pakistan. Int Breastfeed J. (2018) 13:24–35. doi: 10.1186/s13006-018-0171-z, PMID: 29988704 PMC6029391

[28] Raihan MJ Choudhury N Haque MA Farzana FD Ali M Ahmed T. Feeding during the first 3 days after birth other than breast milk is associated with early cessation of exclusive breastfeeding. Matern Child Nutr. (2020) 16:1–8. doi: 10.1111/mcn.12971, PMID: 32048470 PMC7296812

[29] Monge-Montero C van der Merwe LF Papadimitropoulou K Agostoni C Vitaglione P. Mixed milk feeding: a systematic review and meta-analysis of its prevalence and drivers. Nutr Rev. (2020) 78:914–27. doi: 10.1093/nutrit/nuaa016, PMID: 32357372

[30] Tegegne M Sileshi S Benti T Teshome M Woldie H. Factors associated with minimal meal frequency and dietary diversity practices among infants and young children in the predominantly agrarian society of bale zone, Southeast Ethiopia: a community based cross sectional study. Arch Public Heal. (2017) 75:53–63. doi: 10.1186/s13690-017-0216-6, PMID: 29158896 PMC5682638

[31] Patel A Pusdekar Y Badhoniya N Borkar J Agho KE Dibley MJ. Determinants of inappropriate complementary feeding practices in young children in India: secondary analysis of National Family Health Survey 2005-2006. Matern Child Nutr. (2012) 8:28–44. doi: 10.1111/j.1740-8709.2011.00385.x, PMID: 22168517 PMC6860525

[32] Webb P Block S. Nutrition knowledge and parental schooling as inputs to child nutrition in the long and short run. 3, Nutrition Working Paper. (2003). Report No. 3. Available at: http://pdf.usaid.gov/pdf_docs/Pnade922.pdf (Accessed October 27, 2020).

[33] Talukder A. Risk factors associated with wasting among under-5 children residing in urban areas of Bangladesh: a multilevel modelling approach. J Public Heal From Theory to Pract. (2021) 29:525–31. doi: 10.1007/s10389-019-01163-4, PMID: 39670195

[34] Mueni KA. Maternal knowledge on complementary feeding practices and nutritional status of children 6–23 months old, attending Kahawa west public health Centre, Nairobi country School of Applied Human Sciences, Kenyatta University (2014).

[35] Meshram II Laxmaiah A Venkaiah K Brahmam GNV. Impact of feeding and breastfeeding practices on the nutritional status of infants in a district of Andhra Pradesh, India. Natl Med J India. (2012) 25:201–6. doi: 10.12944/CRNFSJ.8.3.17 PMID: 23278776

[36] Anin SK Saaka M Fischer F Kraemer A. Association between infant and young child feeding (IYCF) indicators and the nutritional status of children (6–23 months) in northern Ghana. Nutrients. (2020) 12:2565–82. doi: 10.3390/nu12092565, PMID: 32847027 PMC7551146

[37] Reinbott A Kuchenbecker J Herrmann J Jordan I Muehlhoff E Kevanna O. A child feeding index is superior to WHO IYCF indicators in explaining length-for-age Z-scores of young children in rural Cambodia. Paediatr Int Child Health. (2015) 35:124–34. doi: 10.1179/2046905514Y.0000000155, PMID: 25226288 PMC4462840

[38] Tessema M Belachew T Ersino G. Feeding patterns and stunting during early childhood in rural communities of Sidama, South Ethiopia. Pan Afr Med J. (2013) 14:75–86. doi: 10.11604/pamj.2013.14.75.1630 23646211 PMC3641921

[39] Chowdhury TR Chakrabarty S Rakib M Afrin S Saltmarsh S Winn S. Factors associated with stunting and wasting in children under 2 years in Bangladesh. Heliyon. (2020) 6:e04849. doi: 10.1016/j.heliyon.2020.e04849, PMID: 32984587 PMC7492816

[40] Wali N Agho KE Renzaho AMN. Wasting and associated factors among children under 5 years in five south Asian countries (2014–2018): analysis of demographic health surveys. Int J Environ Res Public Health. (2021) 18:4578–95. doi: 10.3390/ijerph18094578, PMID: 33925898 PMC8123503

[41] Menon P Bamezai A Subandoro A Ayoya MA Aguayo VM. Age-appropriate infant and young child feeding practices are associated with child nutrition in India: insights from nationally representative data. Matern Child Nutr. (2015) 11:73–87. doi: 10.1111/mcn.12036, PMID: 23557463 PMC6860327

